# FokI-RYdCas9 Mediates Nearly PAM-Less and High-Precise Gene Editing in Human Cells

**DOI:** 10.3390/cimb46050248

**Published:** 2024-04-27

**Authors:** Di Li, Yaqi Cao, Long Xie, Chenfei He, Danrong Jiao, Mengxue Ma, Zhenrui Zuo, Erwei Zuo, Xiaogan Yang

**Affiliations:** 1 Guangxi Key Laboratory of Animal Breeding, Disease Control and Prevention, College of Animal Science & Technology, Guangxi University, Nanning 530004, China; 2 Shenzhen Branch, Guangdong Laboratory for Lingnan Modern Agriculture, Key Laboratory of Synthetic Biology, Ministry of Agriculture and Rural Affairs, Agricultural Genomics Institute at Shenzhen, Chinese Academy of Agricultural Sciences, Shenzhen 518000, China

**Keywords:** FokI-RYdCas9, fdCas9, PAM-less, high-precise gene editing

## Abstract

The demand for high-precision CRISPR/Cas9 systems in biomedicine is experiencing a notable upsurge. The editing system fdCas9 employs a dual-sgRNA strategy to enhance editing accuracy. However, the application of fdCas9 is constrained by the stringent requirement for two protospacer adjacent motifs (PAMs) of Cas9. Here, we devised an optimized editor, fRYdCas9, by merging FokI with the nearly PAM-less RYdCas9 variant, and two fRYdCas9 systems formed a dimer in a proper spacer length to accomplish DNA cleavage. In comparison to fdCas9, fRYdCas9 demonstrates a substantial increase in the number of editable genomic sites, approximately 330-fold, while maintaining a comparable level of editing efficiency. Through meticulous experimental validation, we determined that the optimal spacer length between two FokI guided by RYdCas9 is 16 base pairs. Moreover, fRYdCas9 exhibits a near PAM-less feature, along with no on-target motif preference via the library screening. Meanwhile, fRYdCas9 effectively addresses the potential risks of off-targets, as analyzed through whole genome sequencing (WGS). Mouse embryonic editing shows fRYdCas9 has robust editing capabilities. This study introduces a potentially beneficial alternative for accurate gene editing in therapeutic applications and fundamental research.

## 1. Introduction

With continuous advancements in gene editing technology, CRISPR/Cas9 has emerged as one of the most extensively employed tools in gene editing [1,2]. However, safety concerns associated with this tool have gradually heightened in bio-pharmaceutical applications [3,4].The activation of Cas9 protein for the cleavage of the target DNA is contingent upon the presence of a protospacer adjacent motif (PAM) sequence on the sgRNA-matched target DNA sequence. The utilization of a single sgRNA to direct Cas9 in recognizing the target site for genetic modification is frequently constrained by RNA–DNA mismatches, and the conventional Cas9 induces some off-target effects, particularly a close similarity of 10 base pairs following the sgRNA sequence [5,6,7]. To mitigate off-target effects, researchers have developed various high-fidelity Cas9 variants such as HFCas9 [8], eCas9 [9], HypaCas9 [10], or other variants. These variants involve the mutations of amino acids within the catalytic region or the DNA binding pocket of the Cas9 protein, aiming to impose stricter catalytic conditions, such as tighter DNA binding, thereby increasing the specificity of gene editing. However, these Cas9 variants reportedly exhibit favorable editing activity, albeit with varying degrees of reduced editing efficiency [11,12,13]. The reduction in targeting efficiency may be attributed to structural modifications or the weakened DNA binding ability of the Cas9 enzyme.

Furthermore, a strategy known as “dual sgRNAs” could be employed to diminish off-target effects. This strategy necessitates the correct positioning of both left and right sgRNAs for DNA cleavage to occur, thereby enhancing editing specificity. This approach has been employed in two implementation strategies. One involves two sgRNAs guiding nCas9 for cutting the target region [14], while the other includes FokI endonuclease-fused dCas9 (fdCas9) [15,16]. In the latter case, dCas9 is responsible for searching the target sequence, and cleavage is executed by the dimerized FokI enzyme. Before the study of fdCas9, editing tools like TALEN and ZFN utilized dimerized FokI for gene editing [17]. Importantly, dCas9 could not cleave the genome, and off-target effects from fdCas9 theoretically occurred only when both sgRNAs had mismatches in proximity. When utilizing fdCas9 for gene targeting, DNA cleavage can only occur when both sgRNAs guide the FokI molecules into the correct positions, and the dimerized FokI is able to cleave the gene. However, the available target sites are significantly limited due to the requirement of a protospacer adjacent motif (PAM) sequence. Specifically, the presence of an ′NGG′ PAM at both ends of the targeting sequence is necessary.

In recent years, efforts to expand the repertoire of PAM motifs have led to the engineering of various Cas9 variants. The emergence of gene nucleases, such as XCas9, has broadened the spectrum of target sites [18]. Notably, RYCas9 [19], a specific variant, showcases a remarkable nearly PAM-less feature, enabling it to cleave diverse genomic sequences. RYCas9 demonstrates higher editing efficiency with an NRN (R = A/G) PAM compared to NYN (Y = C/T). However, the efficacy of editing through the fusion of the FokI enzyme and deactivated RYCas9 remains unknown, necessitating further evaluation to assess both on-target and off-target effects.

In this study, we successfully established a novel gene-editing tool, termed fRYdCas9, by combining the FokI endonuclease with the nearly PAM-less RYdCas9 variant. This tool presented a much more flexible PAM demand than the reported high-fidelity Cas9 version, while demonstrating precise editing characteristics. We have confirmed that fRYdCas9 exhibits a 330-fold increase in available targeting loci compared to fdCas9 across the entire human genome. Additionally, our findings demonstrate that fRYdCas9 displays comparable editing efficacy to fdCas9. Notably, optimal editing efficiency for fRYdCas9 was observed with a spacer length of 16 base pairs. Importantly, utilizing library screening and whole-genome sequencing (WGS), we discovered that fRYdCas9 demonstrates a remarkable ability to recognize target sites without strict PAM requirements and without showing any preference for specific motifs. This characteristic significantly reduces the occurrence of sg-dependent off-target effects. We also confirmed the fRYdCas9 has robust editing capabilities in mouse embryos. In conclusion, our study indicates the high-fidelity and efficient DNA editing potential of fRYdCas9. It could provide an additional, safer choice for future gene therapy or other applications, especially in cases where other high-fidelity Cas9 variants are unable to perform editing at the desired target site.

## 2. Materials and Methods

### 2.1. Plasmid Construction

The Cas9 construct was based on the PX330 plasmid, generously provided by Feng Zhang (Addgene plasmid #42230). CMV and mCherry were inserted into the Cas9 plasmid. RYCas9 construction followed literature protocols, and dCas9 and dRYCas9 were obtained by introducing two-point mutations, D10A and H840A. Subsequently, FokI was inserted into them to generate fdCas9 and fRYdCas9, provided by David Liu (in addition to gene plasmid #42230). For sgRNA expression plasmids, sgRNA oligos were annealed and inserted before the scaffold (cloned from PX330) by T4 ligations. All plasmids were extracted following the E.Z.N.A plasmid kit manuals. 

For library constructions, oligonucleotides were synthesized, and PCRs were performed (Table S6). The library’s oligonucleotides were cloned into a MluI- and XbaI-digested backbone of the lentiviral plasmid. The Gibson assembly method was employed for library construction. After transduction for 20 h, all DH5α colonies were collected, and plasmids were extracted.

### 2.2. Cell Culture, Transfection, and Genotyping

HEK293T and K562 cells were cultured in DMEM (high glucose) medium supplemented with 10% fetal bovine serum (FBS) and 100 U/mL penicillin/streptomycin (Meilunbio, Dalian, China). The cells were incubated at 37 °C in a humidified atmosphere with 5% CO_2_. For transfection, we co-transfected 2 ug of the editor plasmid and 1 ug sgRNA into HEK293T cells using poly-ethyleneimine (PEI, Polyscience, Warrington, PA, USA) following the manufacturer’s protocols in 12-well plates. Positive cells were isolated by flow cytometry 48 h after transfection, and genomic DNA was extracted using the Crude DNA Extraction Kit (catalog number P072, Vazyme, Nanjing, China). The genotyping of transfected cells was determined using gene-specific primers through nested PCR. In the first round of PCR amplification, Extaq (Takara, Shiga, Japan) was activated at 95 °C for 3 min, then for 30 cycles, with denaturation at 95 °C for 30 s, annealing at 55 °C for 30 s, with an extension at 72 °C for 1 min and a final extension at 72 °C for 5 min after the cycles. The second round of PCR was performed with inner nested primers through the same PCR program. The PCR products were purified for Sanger sequencing or next-generation sequencing. The genotyping identification primers used in this study are listed (Table S7). Next-generation sequencing data were analyzed using CRISPResso2 (version 2.2.7). 

### 2.3. Lentiviral Vector Production and Transduction

For lentiviral production, we collected supernatants containing lentiviral particles 48 h after transfecting HEK293T cells with 30 μg library lentiviral vector, 22.5 μg of psPAX2, and 15 μg of pMD2.G in a 15 cm dish. For the lentiviral transduction of HEK293T cells, the library cell lines were incubated with the lentiviral supernatant. The HEK293T cells, stably expressed in the library, were generated through lentiviral transduction at an MOI of 0.3. This was followed by the selection of EGFP-positive cells using FACS sorting. The library cells were then transfected with editor and sgRNA vectors, and double-positive cells (mCherry+ EGFP+) were sorted out by FACS. Genomic DNA was extracted from the sorted cells using the DNeasy Blood and Tissue Kit (catalog number 69504, Qiagen, Dusseldorf, Germany). PCR products were purified using the Universal DNA Purification Kit (TIANGEN, Beijing, China) following the instructions. The PCR products were then ligated to adapters, and sequencing was performed on the Illumina HiSeq X Ten platform.

### 2.4. Data Analysis for the PAM Library and Edited Window Library

In processing the PAM library data, we summarized the motifs based on the read-editing efficiency. The context plot was generated using the ggseqlogo package in the R software (version 4.2.3). To determine the editing efficiency, we extracted the reads with editing efficiency and calculated the proportion of these edited reads under the same barcode for both ends. We then classified them based on their PAM types. For the editing window-related library, we extracted the editing windows from the edited reads and performed the analysis. Similarly, for context plotting, we used the ggseqlogo package in the R software (version 4.2.3). Regarding editing efficiency, we determined it by calculating the proportion of edited reads to all reads under the same barcode.

### 2.5. Off-Target Detection by Whole Genome Sequencing (WGS)

We sorted individual K562 cells into 96-well plates, and subsequently, cell clones were cultured until confluence was achieved. These single clones of K562 cells were then allocated into three experimental groups. The transfection of the editor and sgRNA was performed using the Nucleofector^®^4D. WGS was executed with an average coverage of 50×, employing the BGI DNBSEQ-T7 platform.

### 2.6. fRYdCas9 Could Be Used in Mouse Embryo Editing

For fRYdCas9 mRNA, we linearized fRYdCas9 plasmids as templates, including a T7 promoter by a restriction enzyme for IVT processes. MESSAGE mMACHINE T7 ULTRA kit (Life Technologies, Waltham, MA, USA) was used for fRYdCas9 mRNA generation. For the IVT of sgRNA, The T7 promoter was added to the sgRNA template by the PCR amplification of px330 (a gift from Feng Zhang; Addgene plasmid #42230), using the primers listed below (Table 1). The T7-sgRNA PCR product was purified and used as the template of IVT using the MEGA shortscript T7 kit (Life Technologies, Waltham, MA, USA). All IVT products of mRNA and SgRNA were purified using the MEGA clear kit (Life Technologies, Waltham, MA, USA) and eluted in RNase-free water.

During the injection, embryos were transferred into a droplet of M2 medium containing 5 μg/mL cytochalasin B (CB), and the FemtoJet microinjector (Eppendorf, Hamburg, Germany) was set to a constant flow. The injected embryos were cultured in KSOM medium with the amino acid at 37 °C under 5% CO_2_ for 2 h and then transferred into the oviducts of pseudo-pregnant ICR females at 0.5 dpc.

For embryo genotyping, in E4.5, a single blastocyst was transferred into 4 µL of medium and was crudely extracted, according to the manufacturer’s instructions, using the Crude DNA Extraction Kit (catalog number P072, Vazyme, Nanjing, China). The nested PCR of targeting loci was performed for embryo cell genotyping. In the first round of PCR amplification, Extaq (Takara, Shiga, Japan) was activated at 95 °C for 3 min, then PCR was performed for 30 cycles, at 95 °C for 30 s, 55 °C for 30 s, and 72 °C for 1 min, and a final extension was carried out at 72 °C for 5 mins after the cycles. The second round of PCR was performed using the same program with an inner nested primer. The PCR products were purified for Sanger sequence genotyping.

### 2.7. Statistical Analysis

For all statistical analyses in this study, we utilized R software (version 4.2.3). All tests were two-sided, and significance levels were denoted as * *p* < 0.05, ** *p* < 0.01, *** *p* < 0.001, and **** *p* < 0.0001.

## 3. Results

### 3.1. Increased Potential Target Sites for fRYdCas9

**Figure 1:**
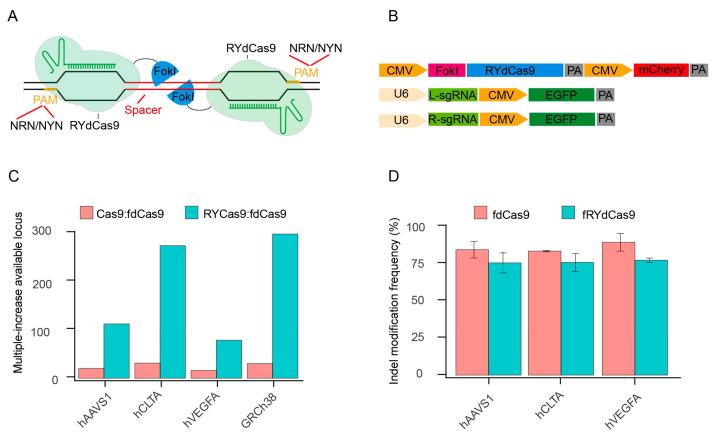
fRYdCas9 significantly enhances the potential target sites. (**A**) Schematic diagram of fRYdCas9. Two FokI nuclease monomers (blue) are coupled to RYdCas9 (light green) and bound to sgRNAs (dark green) to separate loci within the target sites. Only FokI-dCas9 monomers that are bound closely can be fused into a catalytically active FokI nuclease dimer, which subsequently initiates the cleavage of double-stranded DNA. (**B**) Structure diagram of the fRYdCas9 and sgRNAs vectors. The fRYdCas9 is assembled by CMV, FokI nuclease, PYdCas9, and mCherry. The left and right sgRNA vectors are labeled with EGFP. fRYdCas9 or fdCas9 and two sgRNA vectors were co-transfected to HEK293T. Cas9 and fRYdCas9 with one-sided sgRNA served as the controls. (**C**) The ratio of the available loci for Cas9 to fdCas9 and fRYdCas9 to fdCas9 was predicted by the screening of the relevance sequence. Three endogenous genomic targets (*hAAVS1*, *hCLTA1*, and *hVEGFA*) and the human genome (GRCh38) were examined. (**D**) The indel modification frequency of the fRYdCas9 targeting *hAAVS1*, *hCLTA1*, and *hVEGFA* sites via high throughput sequencing.

To characterize fRYdCas9, we constructed it based on the structure of fdCas9 and vectors expressing independent sgRNAs (Figure 1A,B). As FokI forms a dimer to facilitate DNA cleavage, the left and right sgRNAs (L- and R-sgRNA) are required to guide the RYdCas9/dCas9-FokI complex in fRYdCas9 and fdCas9. For the PAM restriction, fRYdCas9 employs NRN/NYN like RYCas9, while fdCas9 uses two NGG PAMs (Figure S1A). Based on the distinct PAM usage rules, we observed that the ratio of available targeting loci for Cas9 to fdCas9 and fRYdCas9 to fdCas9 was approximately 30 and 100–300 times more, respectively (Figure 1C and Table S1).

To verify whether fRYdCas9 could be employed for gene editing, we selected several endogenous genomic targets (*hAAVS1*, *hCLTA*, and *hVEGFA*) for fRYdCas9 (Table S2). Editor and sgRNA vectors were separately labeled with mCherry and EGFP, and the double-positive cells were sorted by flow cytometry (Figure 1B and Figure S1B). The indel modification frequency of fRYdCas9 was comparable to that of fdCas9 by deep sequencing (Figure 1D). Sanger sequencing shows that the fRYdCas9 and fdCas9 possess similar DNA cleavage effects at these three tested loci. Furthermore, fRYdCas9 exhibited no cleavage activity when one side sgRNA was present, while Cas9 can be independently guided by single-sided sgRNA to induce double-strand breaks (DSB) (Figure S1C). Additionally, the editing windows of fRYdCas9 and fdCas9 were similar at the hVEGFA gene (Figure S1D) and other loci. These results indicate that fRYdCas9 exhibits comparable editing efficiency and characteristics to fdCas9.

### 3.2. Validation of Spacer Length for fRYdCas9

**Figure 2:**
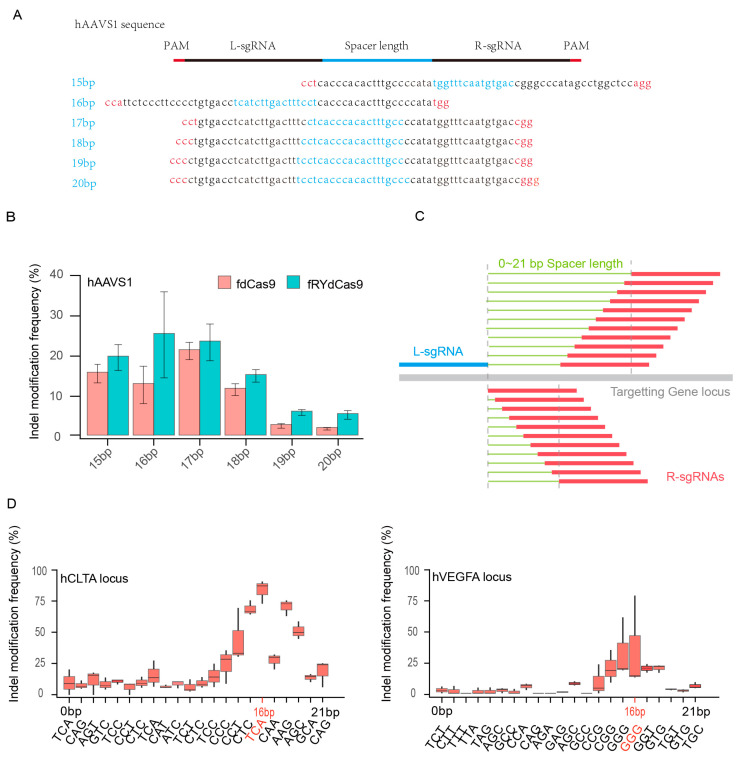
The evaluation of spacer length for fRYdCas9. (**A**) Schematic diagram showing the spacer lengths of 15–20 bps for fRYdCas9 at the *hAAVS1* site. (**B**) Indel modification frequency with increasing spacer lengths for fRYdCas9 at the *hAAVS1* site. fRYdCas9 with various spacer lengths and two sgRNAs vectors were co-transfected to HEK293T. fRYdCas9 fdCas9 served as the control. (**C**) Schematic of the gradually increasing spacer length of 0–21 bps for fRYdCas9. (**D**) Indel modification frequency by varying spacer lengths for fRYdCas9 at the *hCLTA1* or *hVEGFA* sites.

To ascertain the potential for FokI dimer formation in fRYdCas9, six pairs of sgRNAs were designed with spacer lengths ranging from 15 to 20 bp, targeting the hAAVS1 locus. The PAM sequence ′NGG′ was utilized for these sgRNAs (Figure 2A and Table S3). Deep sequencing revealed that the frequency of indel modifications in fdCas9 and fRYdCas9 varied with spacer lengths, indicating a correlation between dimer formation and the distance between L-sgRNA and R-sgRNA (Figure 2B). Leveraging the nearly PAM-less feature of RYdCas9, we conducted more rigorous experiments to elucidate the impact of sgRNA distance on FokI dimer formation. L-sgRNA with a fixed NGG PAM for fRYdCas9 was employed, and sgRNAs with distances ranging from 0 to 21 bp were selected (Figure 2C and Table S4). Notably, at both *hVEGFA* and *hCLTA*, the indel modification frequency of fRYdCas9 varied with spacer length distance, with sgRNA pairs at a distance of 16 bp exhibiting the highest editing efficiency as determined by amplicon sequencing (Figure 2D). Compared to the wide range of PAM-spacer lengths previously reported for fdCas9, we provide a more clearly optimized spacer length here, which might allow for better FokI dimerization and improved catalytic cleavage. This suggests that fRYdCas9 significantly reduces PAM requirements compared to fdCas9, allowing for DNA cleavage without the strict NGG PAM. Consequently, a spacer length of 16 bp is applied for dual sgRNAs with fRYdCas9 in subsequent experiments.

### 3.3. fRYdCas9: A Nearly PAM-Less System

**Figure 3:**
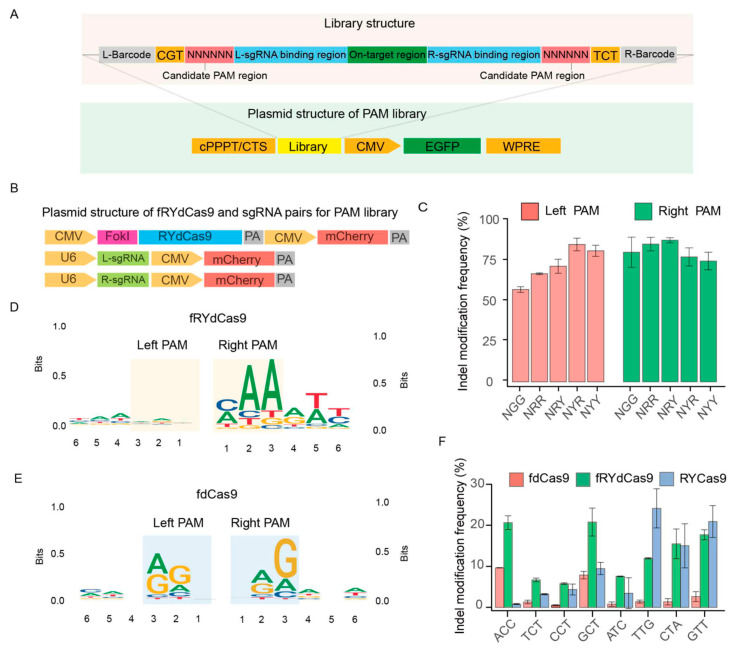
The PAM restriction of fRYdCas9 and RYCas9. (**A**) Structure diagram of the PAM library. The library was assembled by the candidate PAM region, left and right sgRNA (L/R-sgRNA), on-target region, and barcodes. The vector structure for the PAM library is linked with EGFP. The library was packaged into lentivirus, transfected to the HEK293T, and selected by EGFP+ cells. (**B**) Vector structure of editor and sgRNAs. They were linked with mCherry and were co-transfected with the PAM library vector into HEK293T cells. (**C**) Indel modification frequency of fRYdCas9 on various PAM patterns. (**D**) PAM features of fRYdCas9. (**E**) PAM features of fdCas9. (**F**) Indel modification frequency of fRYdCas9 by NYN PAMs.

Building upon the preceding observations, the fRYdCas9 system demonstrated notable editing efficiency even in the absence of the N′GG′ PAM sequence. To further scrutinize the PAM requirements of fRYdCas9, we designed a PAM library to assess its preferences and targeting effects at the hAAVS1 locus. The PAM library construction involved defining a candidate PAM recognition region as ′NNNNNN′, with incorporated barcodes at both ends to aid in identifying on-target sequences and sgRNA binding regions. Additionally, EGFP expression cassettes were linked to the library to facilitate the sorting of integrated library cells (Figure 3A,B). Lentiviral infection was employed to integrate the PAM library into HEK293T cells, and cells with integrated libraries were successfully selected using flow cytometry for subsequent experiments. Sanger sequencing was performed to assess the homogeneity of the library and gene editing efficiency by comparing before and after PAM library transfection (Figure S2). Subsequently, the high-throughput sequencing of the unedited library was conducted to elucidate the relationship between barcodes and PAM sequences. The left and right sgRNAs were initially divided into five groups: NGG, NRR, NRY, NYR, and NYY, to evaluate their respective editing efficiencies. Interestingly, there was no significant difference in the frequency of indel modifications among the NGG, NRR, NRY, NYR, and NYY sgRNA groups in both left and right PAM (Figure 3C).

Following the analysis of edited reads from library sequencing data, we proceeded to examine the left and right PAM context features. In essence, we gathered the 6bp sequences surrounding the left and right sgRNA within the library. Subsequently, we scrutinized their respective biases to construct a motif figure illustrating the bias patterns. In our analysis, we observed an ′NNN′ PAM motif in the left PAM region of the left sgRNA, along with a slight ′AAM′ bias in the right PAM region (Figure 3D). These findings suggest that the editing capacity of fRYdCas9 is more flexible compared to RYCas9. Conversely, when constructing the PAM for fdCas9 (Figure 3E), we noted that fdCas9 exhibited an obvious ′NRG′ and ′RGN′ PAM bias. This suggests that fdCas9 also presents a more flexible PAM bias compared to Cas9 (′NGG′). These data reveal that the fRYdCas9 presented a flexible PAM bias in gene editing. Considering that RYCas9 exhibits weaker cleavage ability when the PAM is NYN compared to NRN, we also validated the cleavage capability of fRYdCas9 at endogenous sites with NYN PAM. We developed a specific one-sided sgRNA PAM sequence to meet the NYN condition for targeting the *hAAVS1* locus. Meanwhile, for the other end, the PAM characteristics are not considered (Table S5). In this study, fRYdCas9 (approximately 25%) exhibited a higher frequency of indel modification in the NYN PAM than that of fdCas9 (2–10%) by high throughput sequencing (Figure 3F), which is similar to the PAM preference of RYCas9.

### 3.4. The fRYdCas9 Exhibits No Context-Dependent Preference in the Targeting Region

**Figure 4:**
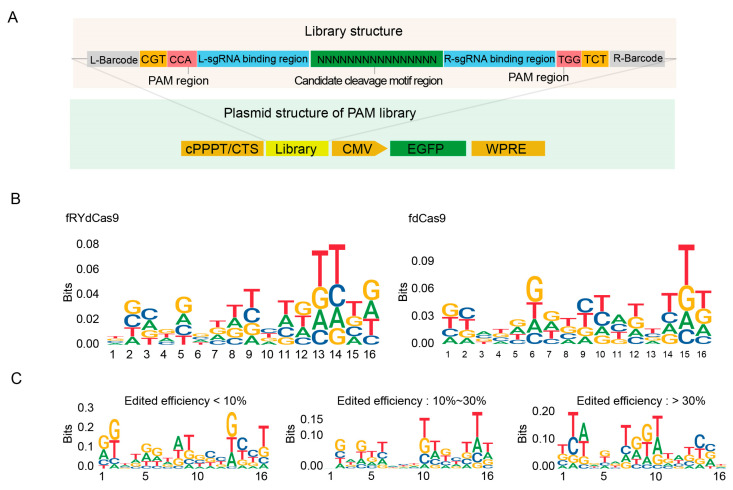
fRYdCas9 shows no specific motif in the editing window. (**A**) Structure diagram depicting the motif library of editing windows. The on-target region is assembled by a random 16-nt library. Vectors were packaged with lentivirus and co-transfected into HEK293T cells. The fdCas9 served as the control. (**B**) The motif probability in the editing window for fRYdCas9. (**C**) Motif preference in the editing window for fRYdCas9 based on editing efficiency.

To determine whether the FokI catalytic dimer in the fRYdCas9 system exhibits any preferences in editing windows, we next utilized a mini-library comprising random 16-nucleotide sequences within the on-target region to assess its context bias. We used the same sgRNAs and barcodes as the PAM library (Figure 4A). Sanger sequencing was performed to assess the homogeneity of the library and gene editing efficiency by comparing the before and after of the targeting region transfection (Figure S3). We extracted the edited reads to analyze the contextual features from the Sanger sequencing data for on-target efficiency, and no distinct contextual feature for fRYdCas9 was found in the 16-nt editing windows (Figure 4B). No specific motif for FokI cleavage was found based on the classification of editing efficiencies (Figure 4C). These results indicate that fRYdCas9 exhibited no context-dependent preference in the on-target region. Taken together with the flexible PAM requirements and a bias towards a more versatile editing context, fRYdCas9 thus holds great promise as a high-fidelity gene editing tool with a wide range of target capabilities for future gene editing applications.

### 3.5. The fRYdCas9 Displays Reduced Off-Target Effects

**Figure 5:**
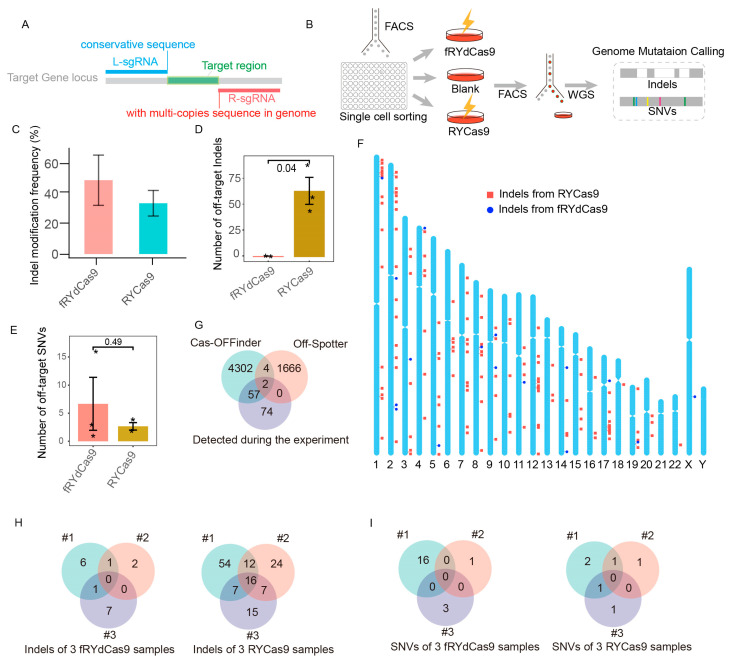
fRYdCas9 exhibits a significant reduction in off-target effects. (**A**) Schematic of sgRNAs simultaneously targeting multi-copy sequences by fRYdCas9. (**B**) Diagram illustrating the setup for the whole-genome sequencing (WGS) process. fRYdCas9 and two sgRNAs vectors were co-transfected to K562 cells. fdCas9 served as the control. (**C**) The indel modification frequency of fRYdCas9 and RYCas9 on the tested locus. (**D**,**E**) The number of indels and single nucleotide variants (SNVs) for fRYdCas9 and RYdCas9. (**F**) The distribution of off-target sites for fRYdCas9 and RYCas9 across the genome. (**G**) Comparison between predicted (Cas-OFFinder and Off-Spotter) and WGS-detected off-target sites for sgRNAs with high off-target effects. (**H**,**I**) Analysis of indels and SNVs between fRYdCas9 and RYdCas9. Data are presented as means ± SEM. * *p* < 0.05, *n* = 3 (Mann–Whitney).

To assess the accuracy of fRYdCas9, we specifically chose a target site with a high probability of off-targets. The target sequence consists of two components: a multiple copies sequence with 1~3 nucleotide differences in the genome (targeted by R-sgRNA), which has potential for off-targets, and a conserved sequence (targeted by L-sgRNA) (Figure 5A). To ensure maximum genomic consistency, we established K562 cell lines from distinct clones and evaluated the potential off-target effects in edited cells using WGS analysis (Figure 5B). 

fRYdCas9 and RYCas9 demonstrated remarkable on-target efficiency (Figure 5C). fRYdCas9 exhibited minimal off-target indels, averaging six, while RYCas9 displayed significantly higher levels of off-target indels, averaging 64 (Figure 5D). By analyzing the off-target sites within repeated samples of fRYdCas9 and eliminating repetitive sequences, we identified only one instance of a regular sequence site exhibiting off-target effects (Figure S4A). Notably, the WGS results reveal that alleles at the off-target sites of fRYdCas9 exhibited low editing frequencies ranging from 15% to 50%, which are significantly lower than those observed at the on-target site (Figure S4B). There was no significant difference in single nucleotide variants (SNV) between fRYdCas9 (average = 7) and RYCas9 (average = 2.5) (Figure 5E). Furthermore, RYCas9’s off-target sites were distributed across multiple chromosomes, minimally overlapping with the anticipated off-target sites by Cas-OFFinder (Figure 5F,G), indicating the challenging predictability of its off-target effects. The analysis of three replicated off-target sequences revealed that mismatches in the sgRNA were the primary cause of off-target effects for RYCas9 but not for fRYdCas9 (Figure 5H and Figure S5A). Additionally, no shared SNVs were observed between fRYdCas9 and RYCas9 (Figure 5I). In addition to off-targets induced by base mismatches, the use of RYCas9 lacking PAM specificity led to off-target effects at a greater number of PAM sequences (Figure S5B). Taken collectively, these findings emphasize the high-fidelity attributes of fRYdCas9 in the gene editing process.

### 3.6. fRYdCas9 Could Be Use in Mouse Embryos Editing

**Figure 6:**
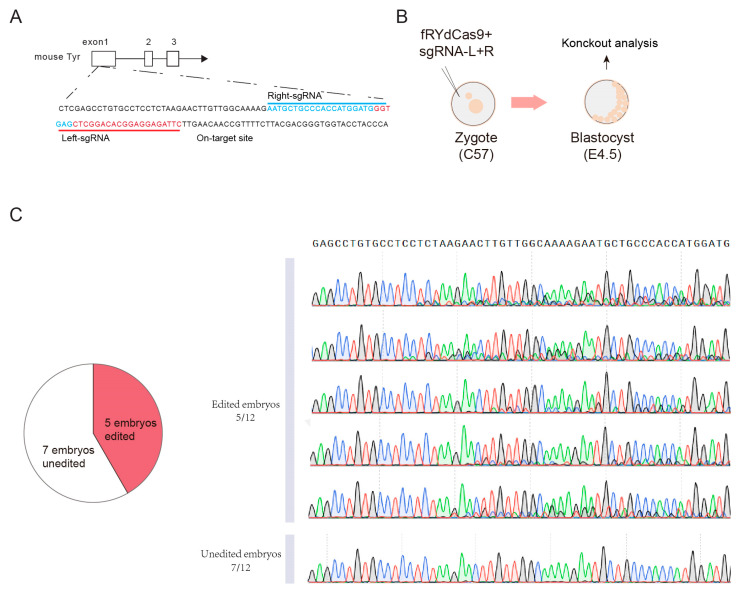
fRYdCas9 exhibits robust mouse embryonic editing capabilities. (**A**) Schematic of on-target site on TRY loci. (**B**) Diagram illustrating the setup for mouse embryo editing. (**C**) Sanger results of edited embryos.

We next conducted experiments to assess the feasibility of using fRYdCas9 for embryonic editing. Our focus was on the TYR gene locus, which is responsible for determining the fur color in mice (Figure 6A). Firstly, we generated mRNA transcripts of fRYdCas9 and sgRNA-pairs specifically targeting the TYR locus through in vitro transcription (IVT) (see Section 2.6). Subsequently, we injected a mixture of fRYdCas9 (100 ηg/μL) and a sgRNA-pair (100 ηg/μL) into the one-cell stage embryos of C57BL/6J mice (Figure 6B). At 4.5 days post-injection, we examined the resulting blastocysts to evaluate the efficiency of on-target gene editing. Remarkably, sanger results show that approximately half of the embryos exhibited noticeable gene modifications (Figure 6C). These data suggest that fRYdCas9 could also be applied in embryo gene editing, indicating its potential capability in constructing animal disease models and other applications.

## 4. Discussion

By replacing the core components of fdCas9 with the PAM-less characteristics of RYdCas9 and the dimerization-mediated cleavage of FokI, we have engineered a highly precise editor, fRYdCas9, which significantly broadens the scope of targetable genomic loci while ensuring high fidelity. Although RYdCas9 has been successfully fused with FokI for gene editing in plants [20], a more comprehensive understanding of its editing characteristics and potential off-target effects is still needed, especially when applied to human cells. In our research, the fRYdCas9 variant demonstrated access to more editing loci, showing a similar editing efficiency to fdCas9. Notably, the fRYdCas9 with a spacer length of 16-nt exhibited the highest editing efficiency. During library screening and WGS analysis, no PAM restriction, targeting motif preference, or minimized off-target effects were observed. The high-precision editor fRYdCas9 holds substantial potential for applications in biomedicine.

In our research, fRYdCas9 exhibited a nearly PAM-less editing characteristic, thereby avoiding the wide-ranging off-target effects of RYCas9. This suggests a more flexible PAM requirement for highly precise gene editing compared to other reported high-fidelity gene editors. The on-target outcomes observed for fRYdCas9 within the library show that FokI endonuclease exhibits no sequence preference for cleavage. Additionally, targeting outcomes from the PAM library demonstrate that fdCas9 can target sites with non-2 NGG PAM types. This observation aligns with existing literature highlighting Cas9’s proficiency in targeting atypical PAMs, such as NGA or NAG [21,22]. Importantly, these findings provide additional confirmation of the robustness of the meticulously constructed library.

In the field of genomic off-target detection, various methods exist, each with its distinct focus [23]. Our study employed a conventional method for detecting off-target effects, involving the generation of monoclonal cell lines, gene editing by editors, and subsequent WGS. To mitigate external noise impact, we established the K562 monoclonal cell line through the isolation of a single-cell clone. Genomic sequencing was then conducted on the edited cells to identify potential off-target effects. In the fRYdCas9 group, we observed that the occurrence of off-target effects did not exhibit any correlation with the sgRNA sequences. This suggests that even in the presence of sgRNAs with a pronounced off-target tendency, fRYdCas9 can effectively constrain their off-target effects. Conversely, for RYCas9, although it has broadened its applicability, it has concurrently led to an increased incidence of off-target effects. We noted a greater number of off-target occurrences specifically associated with the PAM. Notable PAM sequences for off-target sites emerged, including motifs such as CAG, GAG, TAG, and CAA, among others. Our findings reveal that while RYCas9 effectively broadens its targeting scope, offering a beneficial complement for sites beyond the reach of certain Cas9 variants, it also introduces a heightened incidence of off-target effects. Our research indicates that the distinctive sgRNA usage rules of fRYdCas9 are capable of eliminating sgRNA-dependent off-target effects. 

The high-fidelity characteristic of fRYdCas9 may be attributed to its dual-sgRNA binding requirements. This suggests that DNA cleavage by fRYdCas9 requires a 56bp match (comprising sgRNA length and spacer length) in the target region, which is significantly stricter than the binding requirements of wild-type Cas9 or RYCas9, which only require a 20nt match, thereby increasing its editing specificity. Nevertheless, despite its nearly PAM-less and high-fidelity editing features, fRYdCas9 still poses challenges in in vivo therapy delivery due to its large size. At present, it might be more suitable for genetic disease that could be handled at the cellular level, such as hematopoietic stem cell mediated-blood cancer therapy. Further procedures, such as split delivery methods, could be applied to enhance the delivery capability of this system and contribute to more precise gene editing in future applications.

In summary, the incorporation of dimerized FokI into the RYdCas9 variant results in the development of fRYdCas9, which exhibits several notable advantages compared to RYCas9. These advantages include improvements in the PAM feature, targeting motif preference, and reduction in genomic off-target effects. It would present an additional, safer alternative for forthcoming gene therapy or other applications, particularly in scenarios where edits from alternative high-fidelity Cas9 variants could be introduced. This advancement positions fRYdCas9 as a potentially high-fidelity editor in the context of therapeutic applications.

## Figures and Tables

**Table 1:** Primers used for in vitro transcription of sgRNA.

Name	Sequence (5′–3′)
Tyr-L IVT F	TAATACGACTCACTATAGGgcttagaggaggcacaggctcGTTTTA GAGCTAGAAATAG
Tyr-R IVT F	TAATACGACTCACTATAGGgaatgctgcccaccatggatgGTTTTA GAGCTAGAAATAG
sgRNA IVT R	TCTAGCTCTAAAACAAAAAAGCACC

## Data Availability

The raw WGS fastq files used for the analysis have been deposited with the National Center for Biotechnology Information (NCBI) under the accession number PRJNA1054493.

## Supplementary Materials

The following supporting information can be downloaded at: https://www.mdpi.com/article/10.3390/cimb46050248/s1, Figure S1: The construction and assessment of fRYdCas9; Figure S2: The optimization of spacer length for fRYdCas9; Figure S3: The evaluation of PAM restriction for fRYdCas9; Figure S4: Validation results for off-target loci of fRYdCas9; Figure S5: Off-target analysis of fRYdCas9; Table S1: The PAM requirements and potential editing sites for fdCas9, Cas9, and fRYdCas9; Table S2: The left and right (L/R-) sgRNA for fRYdCas9 and fdCas9 at endogenous loci, including hAAVS1, hCLTA, and hVEGFA; Table S3: The 15–20 bp spacer length with ‘NGG‘ PAM for fRYdCas9 targeting gene hAAVS1; Table S4: The increasing spacer lengths for fRYdCas9 at hAAVS1; Table S5: The sgRNA of NYN PAMs for fRYdCas9; Table S6: Oligonucleotides synthesized for library construction; Table S7: Primers used in the experiments.

## References

[1] Hsu P.D., Lander E.S., Zhang F. (2014). Development and Applications of CRISPR-Cas9 for Genome Engineering. Cell.

[2] Doudna J.A., Charpentier E. (2014). The New Frontier of Genome Engineering with CRISPR-Cas9. Science.

[3] Cradick T.J., Fine E.J., Antico C.J., Bao G. (2013). CRISPR/Cas9 Systems Targeting β-Globin and CCR5 Genes Have Substantial off-Target Activity. Nucleic Acids Res..

[4] Lin Y., Cradick T.J., Brown M.T., Deshmukh H., Ranjan P., Sarode N., Wile B.M., Vertino P.M., Stewart F.J., Bao G. (2014). CRISPR/Cas9 Systems Have off-Target Activity with Insertions or Deletions between Target DNA and Guide RNA Sequences. Nucleic Acids Res..

[5] Fu Y., Foden J.A., Khayter C., Maeder M.L., Reyon D., Joung J.K., Sander J.D. (2013). High-Frequency off-Target Mutagenesis Induced by CRISPR-Cas Nucleases in Human Cells. Nat. Biotechnol..

[6] Pattanayak V., Lin S., Guilinger J.P., Ma E., Doudna J.A., Liu D.R. (2013). High-Throughput Profiling of off-Target DNA Cleavage Reveals RNA-Programmed Cas9 Nuclease Specificity. Nat. Biotechnol..

[7] Hsu P.D., Scott D.A., Weinstein J.A., Ran F.A., Konermann S., Agarwala V., Li Y., Fine E.J., Wu X., Shalem O. (2013). DNA Targeting Specificity of RNA-Guided Cas9 Nucleases. Nat. Biotechnol..

[8] Vakulskas C.A., Dever D.P., Rettig G.R., Turk R., Jacobi A.M., Collingwood M.A., Bode N.M., McNeill M.S., Yan S., Camarena J. (2018). A High-Fidelity Cas9 Mutant Delivered as a Ribonucleoprotein Complex Enables Efficient Gene Editing in Human Hematopoietic Stem and Progenitor Cells. Nat. Med..

[9] Slaymaker I.M., Gao L., Zetsche B., Scott D.A., Yan W.X., Zhang F. (2016). Rationally Engineered Cas9 Nucleases with Improved Specificity. Science.

[10] Chen J.S., Dagdas Y.S., Kleinstiver B.P., Welch M.M., Sousa A.A., Harrington L.B., Sternberg S.H., Joung J.K., Yildiz A., Doudna J.A. (2017). Enhanced Proofreading Governs CRISPR–Cas9 Targeting Accuracy. Nature.

[11] Kulcsár P.I., Tálas A., Ligeti Z., Krausz S.L., Welker E. (2022). SuperFi-Cas9 Exhibits Remarkable Fidelity but Severely Reduced Activity yet Works Effectively with ABE8e. Nat. Commun..

[12] Kulcsár P.I., Tálas A., Tóth E., Nyeste A., Ligeti Z., Welker Z., Welker E. (2020). Blackjack Mutations Improve the On-Target Activities of Increased Fidelity Variants of SpCas9 with 5’G-Extended sgRNAs. Nat. Commun..

[13] Jones S.K., Hawkins J.A., Johnson N.V., Jung C., Hu K., Rybarski J.R., Chen J.S., Doudna J.A., Press W.H., Finkelstein I.J. (2021). Massively Parallel Kinetic Profiling of Natural and Engineered CRISPR Nucleases. Nat. Biotechnol..

[14] Ran F.A., Hsu P.D., Lin C.-Y., Gootenberg J.S., Konermann S., Trevino A.E., Scott D.A., Inoue A., Matoba S., Zhang Y. (2013). Double Nicking by RNA-Guided CRISPR Cas9 for Enhanced Genome Editing Specificity. Cell.

[15] Tsai S.Q., Wyvekens N., Khayter C., Foden J.A., Thapar V., Reyon D., Goodwin M.J., Aryee M.J., Joung J.K. (2014). Dimeric CRISPR RNA-Guided FokI Nucleases for Highly Specific Genome Editing. Nat. Biotechnol..

[16] Guilinger J.P., Thompson D.B., Liu D.R. (2014). Fusion of Catalytically Inactive Cas9 to FokI Nuclease Improves the Specificity of Genome Modification. Nat. Biotechnol..

[17] Vanamee É.S., Santagata S., Aggarwal A.K. (2001). FokI Requires Two Specific DNA Sites for Cleavage. J. Mol. Biol..

[18] Hu J.H., Miller S.M., Geurts M.H., Tang W., Chen L., Sun N., Zeina C.M., Gao X., Rees H.A., Lin Z. (2018). Evolved Cas9 Variants with Broad PAM Compatibility and High DNA Specificity. Nature.

[19] Walton R.T., Christie K.A., Whittaker M.N., Kleinstiver B.P. (2020). Unconstrained Genome Targeting with Near-PAMless Engineered CRISPR-Cas9 Variants. Science.

[20] Cheng Y., Sretenovic S., Zhang Y., Pan C., Huang J., Qi Y. (2022). Expanding the Targeting Scope of FokI-dCas Nuclease Systems with SpRY and Mb2Cas12a. Biotechnol. J..

[21] Jiang W., Bikard D., Cox D., Zhang F., Marraffini L.A. (2013). RNA-Guided Editing of Bacterial Genomes Using CRISPR-Cas Systems. Nat. Biotechnol..

[22] Zhang Y., Ge X., Yang F., Zhang L., Zheng J., Tan X., Jin Z.-B., Qu J., Gu F. (2014). Comparison of Non-Canonical PAMs for CRISPR/Cas9-Mediated DNA Cleavage in Human Cells. Sci. Rep..

[23] Tsai S.Q., Joung J.K. (2016). Defining and Improving the Genome-Wide Specificities of CRISPR–Cas9 Nucleases. Nat. Rev. Genet..

