# Supplementary material for: FokI-RYdCas9 Mediates Nearly PAM-Less and High-Precise Gene Editing in Human Cells
Source: Curr Issues Mol Biol. 2024 Apr 27;46(5):4021–34. doi: 10.3390/cimb46050248 (PMC11119187; doi:10.3390/cimb46050248)
Supplement: Supplementary file 1 [file cimb-46-00248-s001.zip › cimb-2952711-supplementary.pdf]

Supplementary Materials

Supplementary Figures

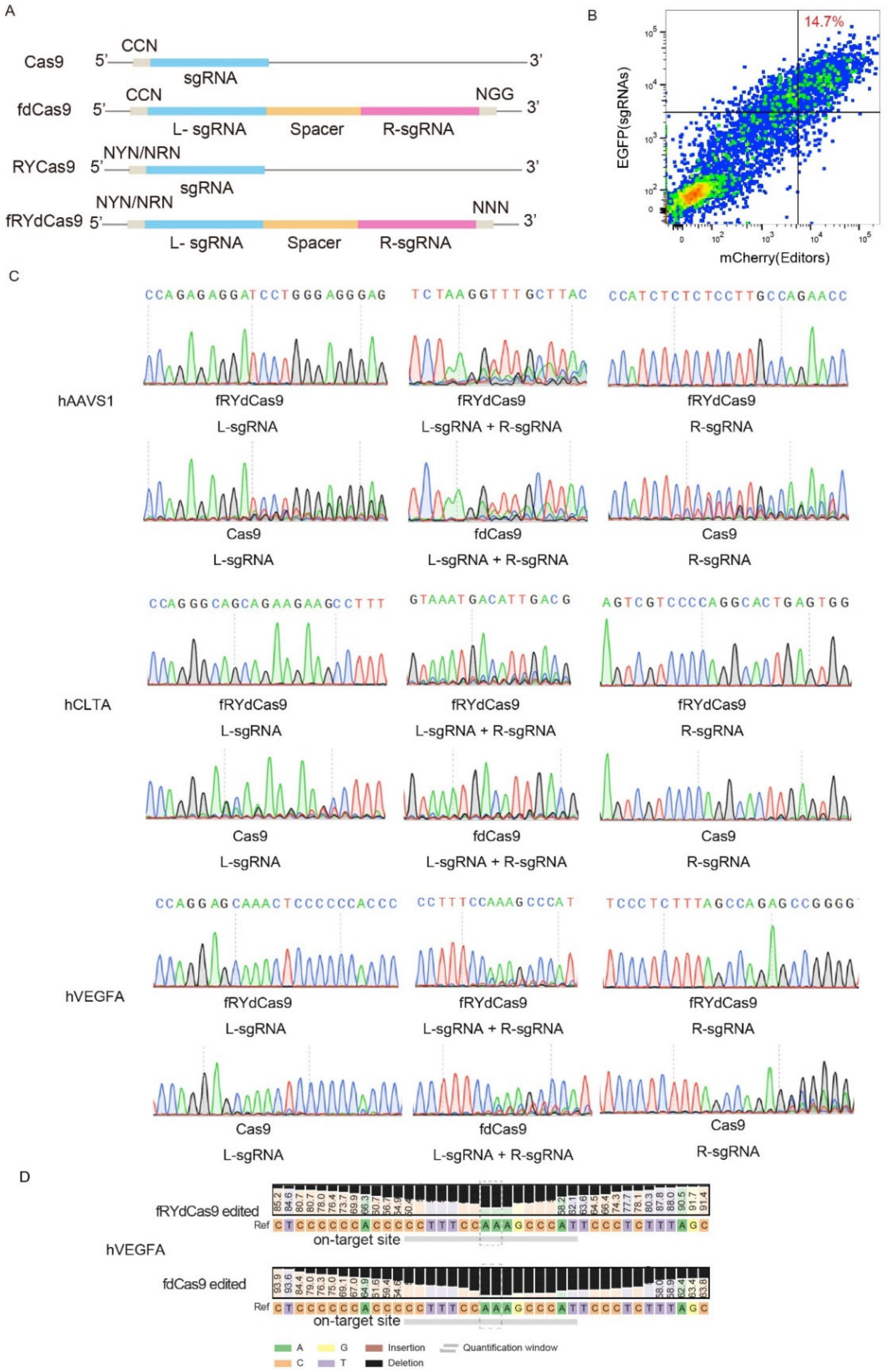

Figure S1. The construction and assessment of fRYdCas9. (**A**) Schematic diagram of sgRNA design for fdCas9, fRYdCas9, and RYCas9. (**B**) Flow cytometry gating strategy for double positive cells after being co-transfected with Editors (linked with mCherry) and sgRNA (linked with EGFP) in HEK293T cells.(**C**) Sanger sequencing chromatograms fRYCas9, fdCas9, and Cas9 at the endogenous targeting sites of hAAVS1, hCLTA1, and hVEGFA. (**D**) Schematic representation of editing windows for fRYCas9 and fdCas9 targeting the hVEGFA gene.

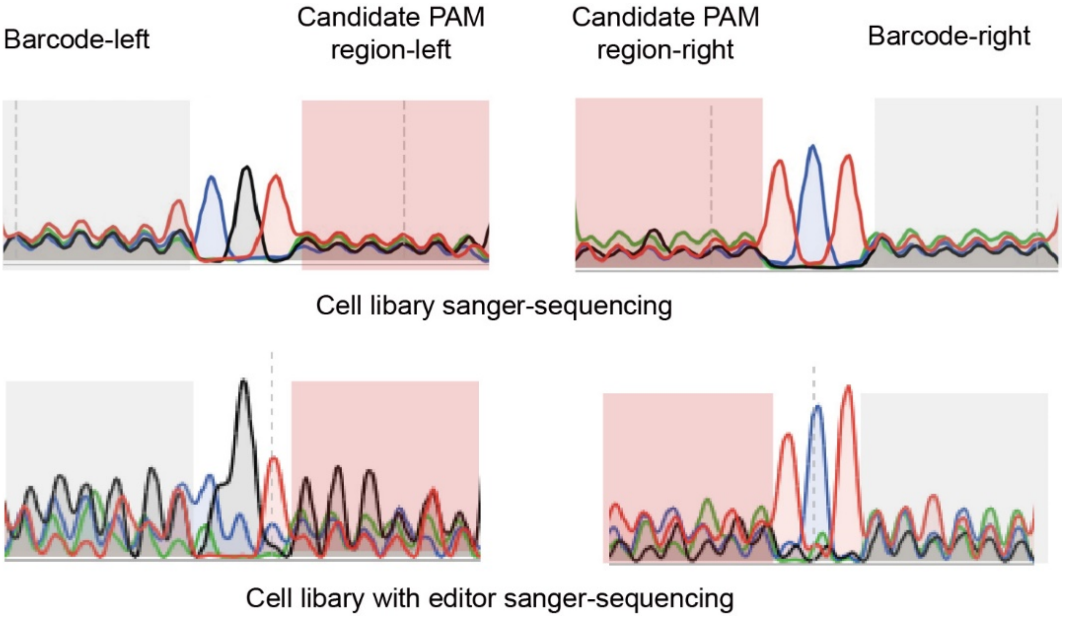

Figure S2. The optimization of spacer length for fRYdCas9. Sanger sequencing chromatograms showing the integration of the PAM library into the genome of HEK293T cells.

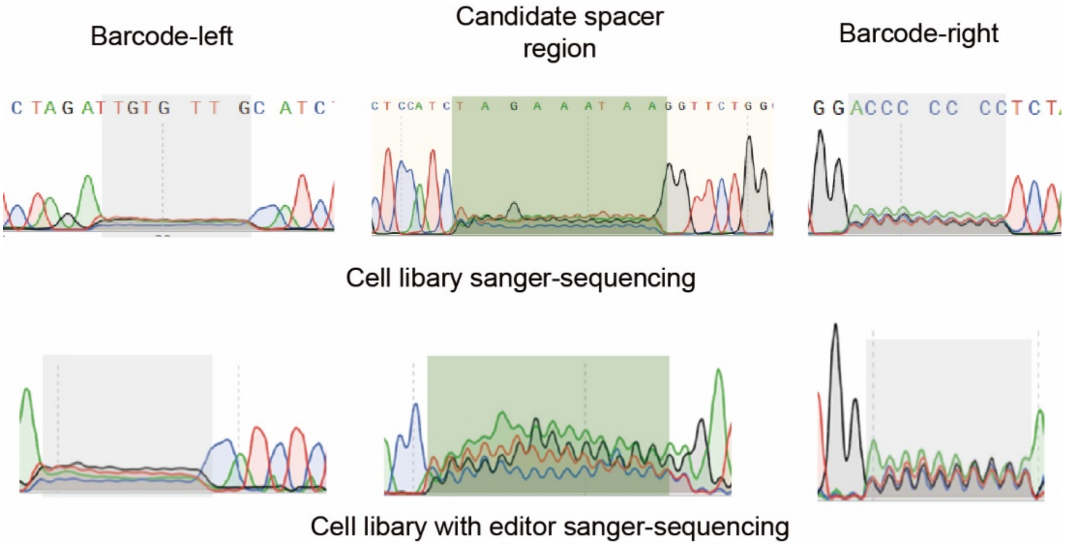

Figure S3. The evaluation of PAM restriction for fRYdCas9. Sanger sequencing chromatograms showing the integration of the editing window library into the genome of HEK293T cells.

A

Sanger validation results for off-target loci of fRYdCas9

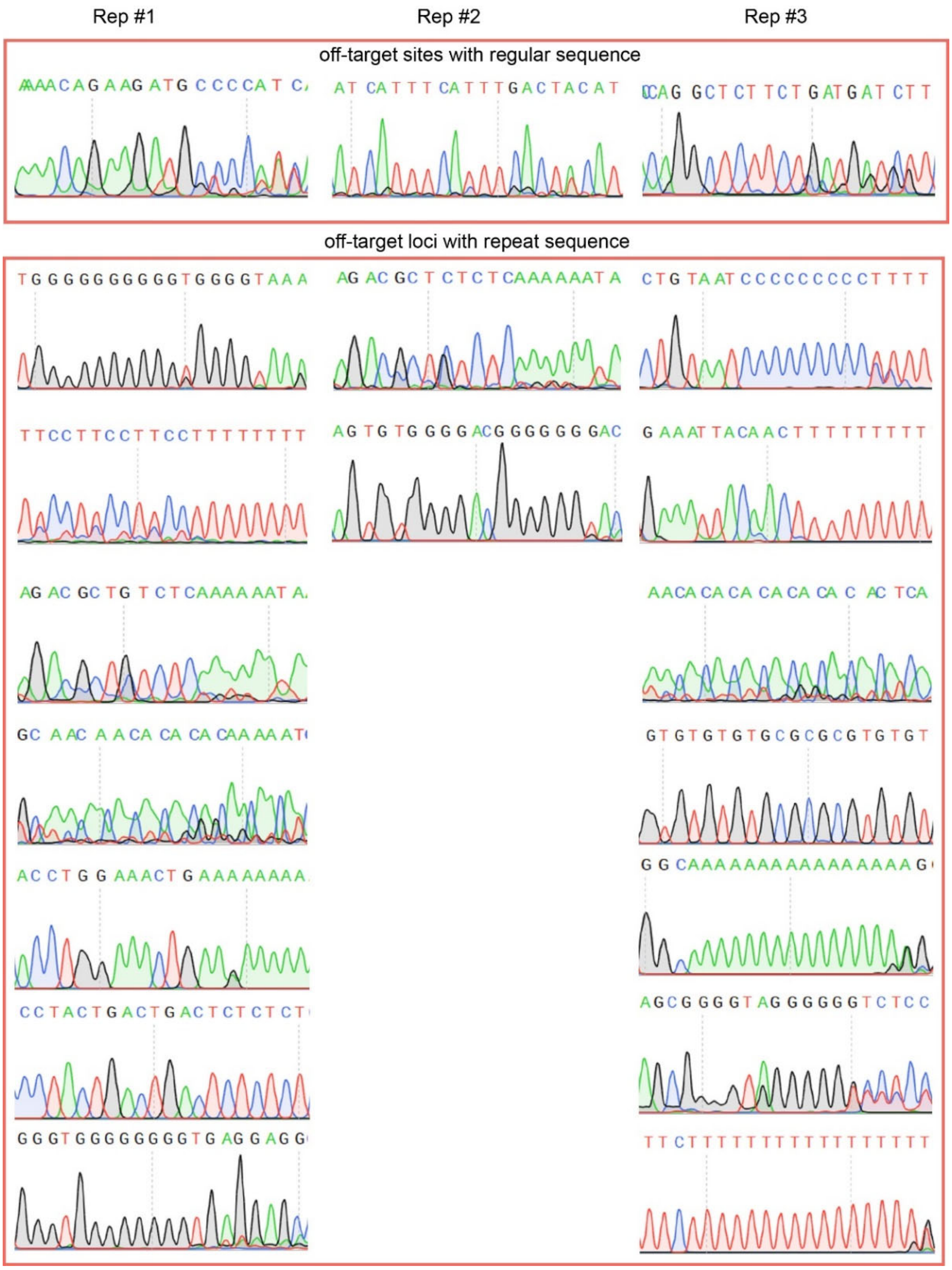

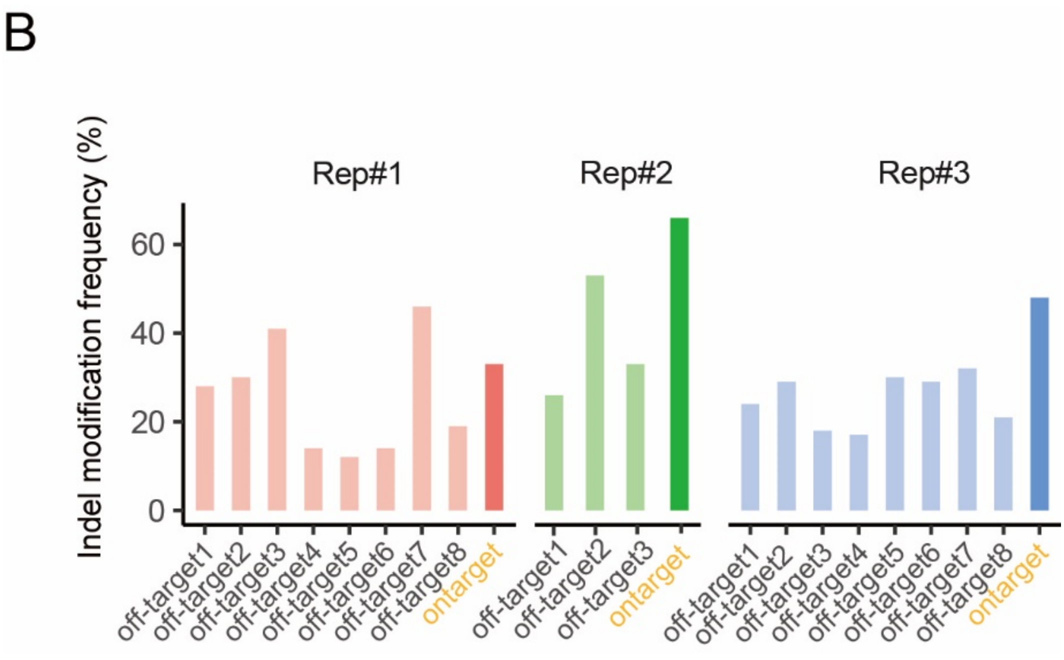

Figure S4. Validation results for off-target loci of fRYdCas9. (**A**) Sanger validation results for off-target loci of fRYdCas9 in three replicates. (**B**) The offtarget loci's indel modification frequency from Whole Genome Sequencing (WGS) data.

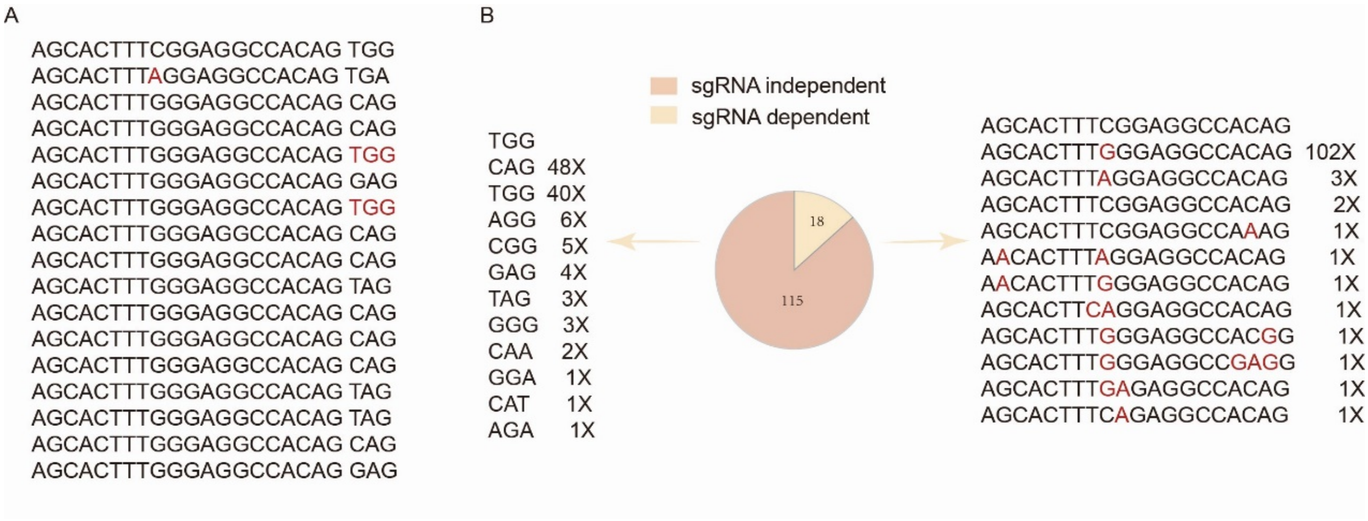

Figure S5. Off-target analysis of fRYdCas9. (**A**) Off-target occurrences of sgRNA observed in all three samples. (**B**) The off-target classification of sgRNAs-dependent and sgRNAs-independent for sgRNAs-dependent.

Supplementary Tables

Table S1. The PAM requirements and potential editing sites for fdCas9, Cas9, and fRYdCas9.

Editors	PAM requirement	hAAVS1(146,664 bp) genebank ID:17	hCLTA(21,186 bp) genebank ID:1211	hVEGFA(, bp) genebank ID:7422	GRCh38
fdCas9	CCN-56*N-NGG	1165	72	185	9078468
Cas9	CCN/NGG	26896	2537	3348	317350282
RYCas9	NRN/NYN	146660	22203	16277	3049314921

Table S2. The left and right (L/R-) sgRNA for fRYdCas9 and fdCas9 at endogenous loci, including hAAVS1, hCLTA, and hVEGFA.

		L-sgRNA	R-sgRNA
locus	hAAVS1	gatggagccagagaggatcc tgg	ggttctggcaaggagagaga tgg
	hCLTA	aaaggcttcttctgctgccc tgg	agtcgtccccaggcactgag tgg
	hVEGFA	gggtgggggagtttgctcc tgg	tccctctttagccagagccg ggg

Table S3. The 15–20 bp spacer length with 'NGG' PAM for fRYdCas9 targeting gene hAAVS1.

Spacer length	L-sgRNA	R-sgRNA
15 bp	tatggggcaaagtgtgggtg agg	cgggcccatagcctggctcc agg
16 bp	ggtcacaggggaagggagaa tgg	cacccacactttgccccata tgg
17 bp	gaaagtcaagatgaggtcac agg	ccatatggtttcaatgtgac cgg
18 bp	aaagtcaagatgaggtcaca ggg	ccatatggtttcaatgtgac cgg
19 bp	aagtcaagatgaggtcacag ggg	ccatatggtttcaatgtgac cgg
20 bp	aagtcaagatgaggtcacag ggg	catatggtttcaatgtgacc ggg

Table S4. The increasing spacer lengths for fRYdCas9 at hAAVS1.

Locus	hCLTA	hVEGFA
left sgRNA	R-sgRNA<br>tggcctgcttgctagacttg ggg	L-sgRNA<br>gggtggggggagtttgctcc tgg
spacer length	sgRNA-R	sgRNA-R
0 bp	aagatgtctcccgcatgcgc tca	cctttccaaagcccattccc tct
1 bp	agatgtctcccgcatgcgct cag	ctttccaaagcccattccct ctt
2 bp	gatgtctcccgcatgcgctc agt	tttccaaagcccattccctc ttt
3 bp	atgtctcccgcatgcgctca gtc	ttccaaagcccattccctct tta
4 bp	tgtctcccgcatgcgctcag tcc	tccaaagcccattccctctt tag
5 bp	gtctcccgcatgcgctcagt cct	ccaaagcccattccctcttt agc
6 bp	tctcccgcatgcgctcagtc ctc	caaagcccattccctcttta gcc
7 bp	ctcccgcatgcgctcagtcc tca	aaagcccattccctctttag cca
8 bp	tcccgcatgcgctcagtcct cat	aagcccattccctctttagc cag
9 bp	cccgcatgcgctcagtcctc atc	agcccattccctctttagcc aga
10 bp	ccgcatgcgctcagtcctca tct	gcccattccctctttagcca gag
11 bp	cgcatgcgctcagtcctcat ctc	cccattccctctttagccag agc
12 bp	gcatgcgctcagtcctcatc tcc	ccattccctctttagccaga gcc
13 bp	catgcgctcagtcctcatct ccc	cattccctctttagccagag ccg
14 bp	atgcgctcagtcctcatctc cct	attccctctttagccagagc cgg
15 bp	tgcgctcagtcctcatctcc ctc	ttccctctttagccagagcc ggg
16 bp	gcgctcagtcctcatctccc tca	tccctctttagccagagccg ggg
17 bp	cgctcagtcctcatctccct caa	ccctctttagccagagccgg ggt
18 bp	gctcagtcctcatctccctc aag	cctctttagccagagccggg gtg
19 bp	ctcagtcctcatctccctca agc	ctctttagccagagccgggg tgt
20 bp	tcagtcctcatctccctcaa gca	tctttagccagagccggggt gtg
21 bp	cagtcctcatctccctcaag cag	ctttagccagagccggggtg tgc

Table S5. The sgRNA of NYN PAMs for fRYdCas9.

PAM	L-sgRNA	R-sgRNA
right-ACC	gccagaacctctaaggtttg ctt	ccaggaaatgggggtgtgtc acc
left-TCT	tctctctccttgccagaacc tct	gggtgtgtcaccagataagg aat
left-CCT	catctctctccttgccagaa cct	gtgtgtcaccagataaggaa tct
left-GCT	tgccagaacctctaaggttt gct	caggaaatgggggtgtgtca cca
right-ATC	atctctctccttgccagaac ctc	ggtgtgtcaccagataagga atc
left-TTG	tcctggagccatctctctcc ttg	cagataaggaatctgcctaa cag
right-CTA	ggagccatctctctccttgc cag	tcaccagataaggaatctgc cta
left-GTT	tccttgccagaacctctaag gtt	aaatgggggtgtgtcaccag ata

Table S6. Oligonucleotides synthesized for library construction.

Library 1	L1 forward primer	ttagtaccgggccctacgcgtNNNNNNcgtNNNNNNggatcctctctggctcca
	L1 substrate	ggatcctctctggctccatcgtaagcaaaccttagaggttctggcaaggagagaga
	L1 reverse primer	tgggctatgaatacctctagaNNNNNNagaNNNNNNtctctctccttgccagaac
Library 2	L2 forward primer	ttagtaccgggccctacgcgtNNNNNNNNNNccaggatcctctctggctcca
	L2 substrate	ggatcctctctggctccatcNNNNNNNNNNNNNNNNNggttctggcaaggagagaga
	L2 reverse primer	tgggctatgaatacctctagaNNNNNNNNNNCCAtctctctccttgccagaacc

Table S7. Primers used in the experiments.

**TableS2 primers**	hAAVS1	F1 gcagcaccaggatcagtg	R1 ggttaatgtggctctggttc
		F2 agaaccgggcaggtcacg	R2 acctcctgttaggcagattc
	hVEGFA	F1 gaagcatccctggacacttc	R1 acgtcctcactctcgaagac
		F2 gtacatgaagcaactccagtccc	R2 cagcttccctgtggtggc
	hCLTA	F1 ggcagttgcttgtgtagcaag	R1 tctccctcctctctgaatgcca
		F2 tcgtccccaggcactgag	R2 atgtagtgtttccacagggtgg
**TableS3 primers**	hAAVS1	F1 acatcatgtcttcttcctgccag	R1 tgcaggcccatatcacagc
		F2 gctgcttgggcctacgaaa	R2 ggtcagatggcagctcctg
**TableS4 primers**	hCLTA	F1 cgtgtctacctgagtctgacct	R1 gcacaggttgaggctactctt
		F2 ggcagttgcttgtgtagcaag	R2 tctccctcctctctgaatgcca
	hVEGFA	F1 gaagcatccctggacacttc	R1 cacagatctattggaatcctggag
		F2 cagtcactccagcctgttg	R2 acgtcctcactctcgaagac
**TableS5 primers**	hAAVS1	F1 gcagcaccaggatcagtg	R1 ggttaatgtggctctggttc
		F2 ctcccaggatcctctctg	R2 tgatattgggtctaaccccacc
